# Performance of metagenomic Next-Generation Sequencing and metagenomic Nanopore Sequencing for the diagnosis of tuberculosis in HIV-positive patients

**DOI:** 10.3389/fcimb.2024.1423541

**Published:** 2024-08-21

**Authors:** Jing Yuan, Lanchun Wang, Wei Zhang, Changgang Deng, Qisui Li, Yamin Meng, Yaokai Chen

**Affiliations:** ^1^ Department of Infectious Diseases, Chongqing Public Health Medical Center, Chongqing, China; ^2^ Medical Center, Key Laboratory of Digital Technology in Medical Diagnostics of Zhejiang Province, Hangzhou, China

**Keywords:** MNPs, MNGs, tuberculosis, HIV-infection, diagnostic performance

## Abstract

**Background:**

Patients who were infected by the *Human Immunodeficiency Virus (*HIV) could have weakened immunity that is complicated by opportunistic infections, especially for *Mycobacterium tuberculosis* (MTB). Notably, the HIV-MTB co-infection will accelerate the course of disease progress and greatly increase the mortality of patients. Since the traditional diagnostic methods are time-consuming and have low sensitivity, we aim to investigate the performance of mNGS (metagenomic Next-Generation Sequencing) and mNPS (metagenomic NanoPore Sequencing) for the rapid diagnosis of tuberculosis in HIV-infected patients.

**Methods:**

The 122 HIV-infected patients were enrolled for the retrospective analysis. All of the patients underwent traditional microbiological tests, mNGS, and (or) mNPS tests. The clinical comprehensive diagnosis was used as the reference standard to compare the diagnostic performance of culture, mNGS, and mNPS on tuberculosis. We also investigate the diagnostic value of mNGS and mNPS on mixed-infection. Furthermore, the treatment adjustment directed by mNGS and mNPS was analyzed.

**Results:**

Compared with the composite reference standard, the culture showed 42.6% clinical sensitivity and 100% specificity, and the OMT(other microbiological testing) had 38.9% sensitivity and 100% specificity. The mNGS had 58.6% clinical sensitivity and 96.8% specificity, and the mNPS had 68.0% clinical sensitivity and 100% specificity. The proportion of mixed-infection cases (88.9%) in the TB group was higher than those in the non-TB group (54.8%) and the mNGS and mNPS are more competitive on mixed-infection diagnosis compared with the traditional methods. Furthermore, there are 63 patients (69.2%) and 36 patients (63.2%) achieved effective treatment after receiving the detection of mNPS and mNGS, respectively.

**Conclusion:**

Our study indicated that mNPS and mNGS have high sensitivity and specificity for TB diagnosis compared with the traditional methods, and mNPS seems to have better diagnostic performance than mNGS. Moreover, mNGS and mNPS showed apparent advantages in detecting mixed infection. The mNPS and mNGS-directed medication adjustment have effective treatment outcomes for HIV-infected patients who have lower immunity.

## 1 Introduction

Acquired Immune Deficiency Syndrome (AIDS) infected by the *Human Immunodeficiency Virus (*HIV) causes human immune dysfunction and is complicated by various serious opportunistic infections, including bacteria, fungi, viruses, and parasites (Di Pasquale et al., 2019; Wang et al., 2019; Xie et al., 2021). In HIV-infected patients, *Mycobacterium tuberculosis* (MTB) is a common opportunistic pathogen that is widely distributed worldwide and was the leading cause of death in a single pathogen-infected disease before the coronavirus (COVID-19) pandemic (Bagcchi, 2023). AIDS patients complicated with tuberculosis (TB) will accelerate the course of AIDS progress, which greatly increases the death rate of patients. According to the reports, about 0.25 million deaths were attributed to TB associated with HIV, and nearly 15% of all global tuberculosis deaths were attributed to multiple drug resistant tuberculosis (MDR-TB) (Global tuberculosis report 2019, 2019). Therefore, it is an even more pressing priority to find accurate and fast diagnostic strategies for TB for HIV patients.

Patients with HIV-TB co-infection have low immunity and their clinical and imaging manifestations are more atypical. Research in South Africa showed that up to 45.8% of HIV-infected patients fail to diagnose TB before death (Bates et al., 2015). The traditional identification strategy of MTB is mainly based on the morphology, growth rate, and biochemical reaction to distinguish MTB and non-tuberculosis mycobacterium (NTM). However, high nutritional requirements and slow growth lead to low sensitivity, cumbersome operation, and time-consuming methods, which are not conducive to early diagnosis and treatment. In recent years, progress has been made in the field of MTB with new molecular assays, such as Xpert MTB/RIF Ultra, line probe assays, and loop-mediated isothermal amplification (TB-LAMP) (World Health Organization, 2008; World Health Organization, 2016; World Health Organization, 2017). However, these assays are still limited by low-throughput, low pathogen detection spectrum that could not completely meet the clinical requirements. Therefore, it is vital to develop a rapid diagnostic method for TB to improve the diagnostic efficiency of TB in HIV-infected people.

Metagenomic next-generation sequencing (mNGS) has become a promising method for diagnosing infectious diseases, on account of culture independence, high throughput, and fast turnaround time (TAT) (Blauwkamp et al., 2019; Wilson et al., 2019; Chen et al., 2020). However, the reading length is short (50-600bp), which affects the efficiency and accuracy of its splicing assembly (Chiu and Miller, 2019; pretermmicrobiota and antimicrobial-resistant pathogens, 2020). By contrast, Given high speed (sequencing speed >400bp/s), long read length, and antibiotic resistance analysis (Kinder et al., 2001; Alonso et al., 2011), Metagenomic nanopore sequencing (mNPS), which is also called Metagenomic third-generation sequencing, seems more suitable for clinical application (Chiu and Miller, 2019; Leggett et al., 2020).

In this study, we aimed to assess the diagnosis efficiency of mNGS and mNPS for Tuberculosis identification and Mixed-infection analysis in HIV-positive patients and elucidate the technical advantages of the two methods. Our study could provide an important reference for the application and promotion of a pathogen identification detection system in the future.

## 2 Materials and methods

### 2.1 Study design and subjects

The retrospective study enrolled the HIV-infected patients in Chongqing Public Health Medical Center (China) from January 2022 to July 2023. The inclusion criteria of this study were described as follows (1): Patients who were diagnosed with AIDS and were suspected of lower respiratory tract infections (LRTIs) such as fever, cough, coughing of blood, purulent sputum, chest pain, breathlessness or dyspnea (2). Patients whose diagnosis of LRTIs is supported by radiological evidence (3). Patients with completed clinical testing, documented treatment, and clinical outcome (14 days of follow-up) records. The exclusion criteria were (1): Patients have incomplete clinical information (2). Patients who have not completed the culture, mNGS, or mNPS testing. The clinical comprehensive diagnosis of each patient in the study serves as a benchmark for assessing the sensitivity and specificity of the test method. The diagnosis was made by a discussion between two respiratory physicians on the medical team. The HIV infection and tuberculosis was diagnosed according to AIDS diagnosis and treatment guidelines and WHO diagnostic criteria for tuberculosis (AIDS and Hepatitis C Professional Group et al., 2021; WHO consolidated guidelines on tuberculosis: Module 3: Diagnosis – Rapid diagnostics for tuberculosis detection, 2024). The criteria for the improvement of curative effect: the clinical symptoms of the patients were improved, and the imaging lesion absorption and reduction were observed. The criteria for drug adjustment: Adjustment according to “the Sanford guide to antimicrobial therapy”, examination of pathogens and/(or) drug susceptibility results.

### 2.2 Conventional microbiological tests

The bronchoalveolar lavage fluid (BALF) samples were tested using the following methods, including bacterial culture, gram staining, mycobacterial culture, and fungal culture. The acid-fast staining, Gene-Xpert, and T-SPOT assays were conducted for *Mycobacterium tuberculosis* detection. Fungal immunofluorescence staining, silver hexamine staining, galactomannan antigen tests, and *Cryptococcus* capsular polysaccharide antigen were performed for fungi detection.

### 2.3 mNPS platform: DNA extraction, library construction, sequencing and data analysis

The BALF samples were collected and aliquoted for both conventional microbiological tests and mNGS or mNPS tests. The DNA extraction, construction library, and sequencing process under the Nanopore platform were performed as previously described (Gu et al., 2021; Luo et al., 2023; Zhao et al., 2024). Briefly, the DNA was extracted from BALF samples (300μl). Then, the concentration of each sample was calibrated after host DNA depletion. For the mNPS platform, the DNA was then fragmented, ligated, library purification, and PCR amplification (Zhao et al., 2024), according to the manufacturer’s instructions (Oxford Nanopore Technologies, Oxford, UK).

The sequencing was then conducted using the GridION platform (Oxford Nanopore Technologies, Oxford, UK) after library preparation. The original data file was generated by the MinION sequencer. Then, The sequence real-time identification and the low-quality value sequences filtration were conducted by MinKnow software. Furthermore, the host DNA removal process was performed by the Minimap2 software using the human genome reference sequence Hg38. Centri figure v1.0.3 is used for multi-sequence alignment with NCBI (https://www.ncbi.nlm.nih.gov/).

### 2.4 mNGS platform: DNA extraction, library construction, sequencing and data analysis

The DNA was extracted using a DNA extraction and Purification Kit (Genskey Co., Ltd, Beijing, China) according to the manuscript’s instructions. The library construction was conducted using the NEB Next Ultra II DNA Library Prep Kit(New England Biolabs Inc.) (Miller et al., 2019). The prepared library pool was sequenced using the Illumina Nextseq 550 DX sequencer, and about 20 million (M) raw data for each sample would be produced. Subsequently, The low-quality reads, adaptor contamination, and duplicate reads, as well as those shorter than 50 bp were removed. The Burrows-Wheeler Aligner (BWA) was then used to identify the sequence by mapping the Human Reference genome (Genome Reference Consortium Human Build 38, GRCh38) (Li and Durbin, 2009). The filtered data were aligned to the in-house Genome database (Dian Diagnostics Pathogenic Microorganism Genome Database) using BWA software.

### 2.5 Statistical analysis

The numerical variables were showed as mean and standard deviation (SD), and compared by the One-way ANOVA. The nominal variables were described by counts and percentages, and compared by the Chi-square test. The clinical comprehensive diagnosis served as the Composite Reference Standard and compared the diagnostic performance of Culture, OMT (Other Microbiological Tests), mNGS, and mNPS. Based on true positive (TP), true negative (TN), false positive (FP), and false negative (FN) for pathogens detection which served as the Composite Reference Standard, the sensitivity [TP/(TP+FN)], specificity [TN/(TN +FP)], Positive predictive value (PPV) [TP/(TP+FP)], Negative predictive value (NPV) [TN/(TN+FN)], False positive rate (FRP) [FP/(FP+TN)], False Negative rate (FNR) [FN/(TP+FN)] and accuracy [(TP+TN)/(TP+FP+TN+FN)], as well as the corresponding 95% confidence intervals (CI) can be calculated using the reportROC package in R (version 4.2.2). A two-sided P value of less than 0.05 was considered significant for all tests unless indicated otherwise (*P<0.05, **P<0.01, ***P<0.001).

## 3 Results

### 3.1 Clinical characteristics of HIV-infected patients

**Figure 1:**
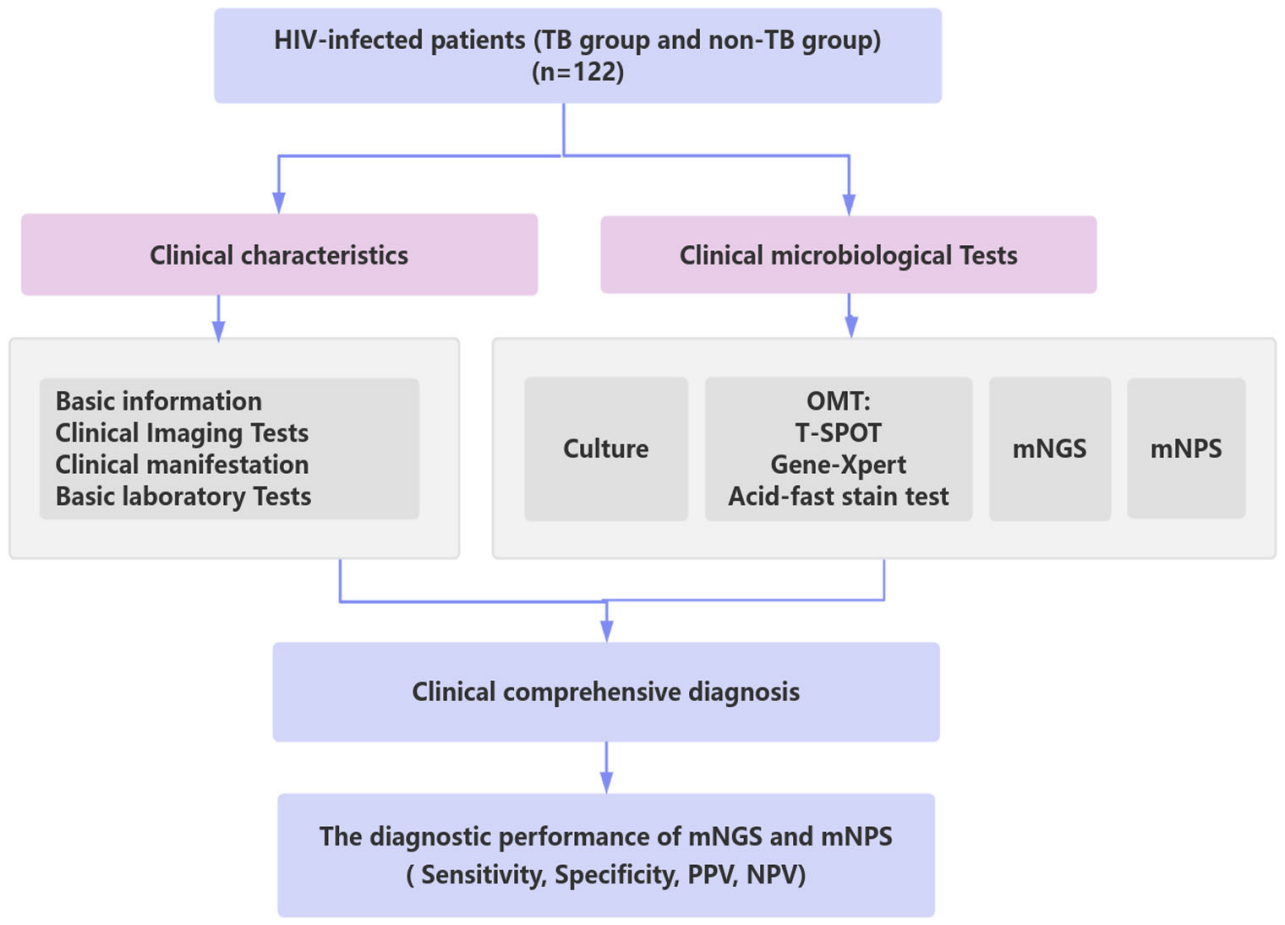
Schematic workflow for TB diagnosis of this study. A total of 122 HIV-infected patients suspected of lower respiratory tract infections (LRTIs) underwent clinical characteristics analysis, traditional microbiological tests, mNGS, and mNPS tests. Based on the clinical comprehensive diagnosis, The diagnostic performance of mNGS and mNPS was evaluated in the enrolled cohort. TB group: the patients were co-infected with HIV and TB. non-TB group: the patients were infected with HIV and without TB.

This study retrospectively analyzed the clinical data of 122 HIV-infected patients who were treated in Chongqing Public Health Medical Center from January 2022 to July 2023. According to the clinical comprehensive diagnosis, the patients were divided into the tuberculosis group (TB group, 58 cases) and non-tuberculosis group (non-TB group, 62 cases), and 2 patients were excluded from the study due to the incomplete clinical information (**Figure 1**). The baseline data are shown in **Table 1**. The Hemoglobin (Hb) (98.71 ± 23.68 VS 112.74 ± 23.31, P=0.001), CD3^+^ T cells (569.21 ± 436.72 VS 843.02 ± 596.09, P=0.005), and CD8^+^ T cells (428.36 ± 341.02 VS 616.06 ± 556.25, P=0.029) were significantly decreased in TB group compared with non-TB group. However, There were no significant differences in age, gender, white blood cells (WBC), neutrophils, lymphocyte, CD4^+^ T cell, Platelets (PLT), packed cell volume (PCV), C-reactive protein (CRP), procalcitonin (PCT), HIV viral load, lactate dehydrogenase (LDH), and the clinical manifestations between the two groups.

**Table 1:** Clinical characteristics of TB and no-TB patients.

Characteristic	TB	non-TB	P-value
Cases	58	62	
Age (years), mean (SD)	52.89 (12.95)	50.94 (15.77)	0.463
Gender (male), n (%)	49 (84.5)	46 (74.2)	0.245
Hb (g/L), mean (SD)	98.71 (23.68)	112.74 (23.31)	**0.001**
WBC (×10^9^/L), mean (SD)	5.64 (2.61)	7.18 (10.60)	0.287
Neutrophil (×10^9^/L), mean (SD)	4.21 (2.57)	3.65 (2.22)	0.212
Lymphocyte (×10^9^/L), mean (SD)	3.27 (17.83)	1.22 (0.85)	0.367
CD3^+^ T cell (cells/μl), mean (SD)	569.21 (436.72)	843.02 (596.09)	**0.005**
CD8^+^ T cell (cells/μl), mean (SD)	428.36 (341.02)	616.06 (556.25)	**0.029**
CD4^+^ T cell (cells/μl), mean (SD)	122.64 (238.80)	184.55 (151.17)	0.09
CD4/CD8, mean (SD)	0.28 (0.33)	0.30 (0.28)	0.741
PLT (×10^9^/L), mean (SD)	195.67 (110.78)	196.31 (105.93)	0.974
PCV (%), n (%)	31.98 (13.21)	33.87 (6.39)	0.318
CRP (mg/L), mean (SD)	57.77 (59.84)	40.27 (57.06)	0.108
PCT (ng/ml), mean (SD)	3.33 (15.03)	0.25 (0.51)	0.118
HIV viral load (Copies×10^5^/ml), mean (SD)	7.2 (21.33)	107 (763)	0.325
LDH (U/L), mean (SD)	267.61 (105.69)	351.34 (449.88)	0.173
Clinical manifestations, n (%)			
Fever	28 (48.3)	28 (45.2)	0.874
Cough	40 (69.0)	42 (67.7)	1
Purulent sputum	25 (43.1)	24 (39.3)	0.818
Breathlessness or dyspnea	12 (21.1)	22 (35.5)	0.124
Lung rales	5 (8.8)	10 (16.1)	0.352
Psychogenic symptoms	2 (3.5)	7 (11.3)	0.209
lymphadenectasis	4 (7.0)	2 (3.2)	0.6
Splenomegaly/Hepatomegaly	5 (8.8)	2 (3.2)	0.371
Skin/oral lesions	3 (5.3)	2 (3.2)	0.923
Chest CT abnormalities	57 (100.0)	61 (98.4)	1
Anti HIV treatment	23 (39.7)	17 (27.9)	0.243

Hb, Hemoglobin; WBC, White Blood cells; PLT, Platelets; PCV, packed cell volume; CRP, C-reactive protein; PCT, procalcitonin; LDH, Lactate dehydrogenase. Bold represents a significant difference.

### 3.2 Detection performances of the mNGS and mNPS on TB infection

**Figure 2:**
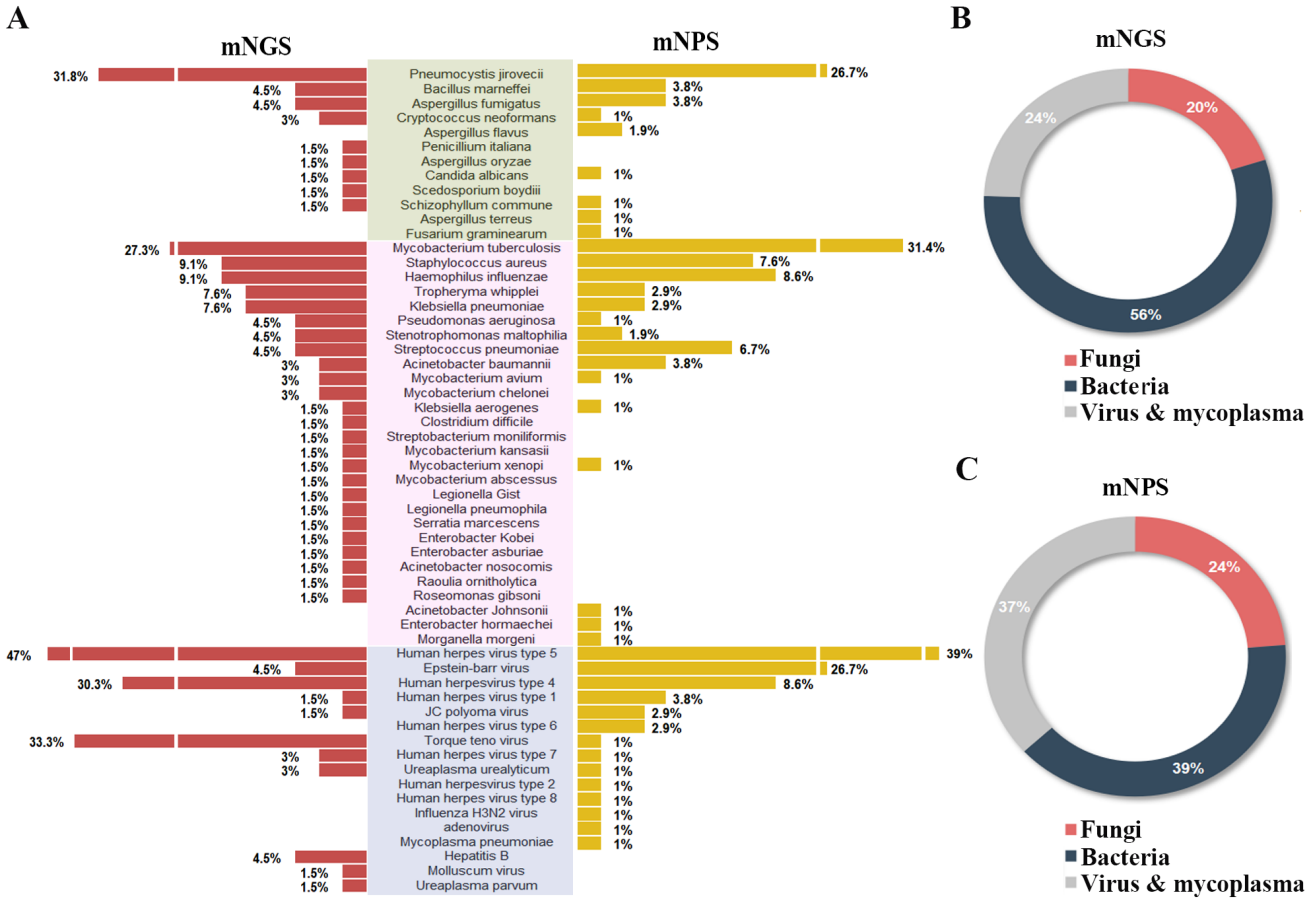
Comparison of the pathogens detection rate of HIV-infected patients on mNGS and mNPS platform. **(A)** The pathogen detection rate of each species on the mNGS and mNPS platform. **(B)** The pie chart of the distribution of detected pathogens by mNGS and **(C)** mNPS. Pathogen categories: Fungi, green background; Bacteria, pink background; Virus & mycoplasma, blue background.

All 120 cases were detected by culture and other microbiological tests (OMT, including Acid-fast stain test, Gene-Xpert, T-SPOT), and 66 cases were detected by mNGS, 105 cases were detected by mNPS. The workflow for Bronchoalveolar Lavage Fluid (BALF) samples using conventional culture, clinical microbiological tests, mNGS, and mNPS are shown in **Figure 2**. Of the 116 cases (4 cases with extrapulmonary tuberculosis were not included), only 19.8% (23/116) of the cases were culture-positive for MTB, and 18.1% (21/116) of the cases were positively detected by OMT. Notably, the positive result was elevated to 30.0% (18/60) and 33.6% (34/101) with the detection of mNGS and mNPS, respectively. Compared with the composite reference standard, the culture showed 42.6% [95% confidence interval (CI), 29.4%–55.8%] clinical sensitivity and 100% [95% CI, 100%–100%] specificity, the OMT had 38.9% [95% CI, 25.9%–51.9%] sensitivity and 100% [95% CI, 100%–100%] specificity. The mNGS had 58.6% [95% CI, 40.7%–76.5%] clinical sensitivity and 96.8% [95% CI, 90.6%–100%] specificity, and the mNPS had 68.0% [95% CI, 55.1%–80.9%] clinical sensitivity and 100% specificity (**Table 2**). The mNGS had 3.2% [95% CI, 1.6%–6.6%] false positive rate(FPR), whereas the FPR of the culture, OMT, mNPS was 0. In addition, The false negative rate (FNR) of culture, OMT, mNGS and mNPS were 57.4% [95% CI, 45.6%–72.2%], 61.1% [95% CI, 49.4%-75.6%], 41.4% [95% CI, 21.6%-66.3%], and 32.0% [95% CI, 21.4%-47.9%] (**Table 3**).

**Table 2:** Diagnostic performance of culture, OMT, mNGS and mNPS in TB and non-TB patients.

Test	TB		Non-TB		Sensitivity (95%CI)	Specificity (95%CI)	PPV (95%CI)	NPV (95%CI)	Accuracy (95%CI)
	+	-	+	-					
Culture	23/54	31/54	0/62	62/62	42.6% (29.4-55.8)	100% (100-100)	100% (100-100)	66.7% (57.1-76.2)	73.3% (72.9-73.6)
OMT	21/54	33/54	0/62	62/62	38.9% (25.9-51.9)	100% (100-100)	100% (100-100)	65.3% (55.7-74.8)	71.6% (71.2-71.9)
mNGS	17/29	12/29	1/31	30/31	58.6% (40.7-76.5)	96.8% (90.6-100)	94.4% (83.9-105)	88.1% (78.3-97.9)	78.3% (77.8-78.9)
mNPS	34/50	16/50	0/51	51/51	68.0% (55.1-80.9)	100% (100-100)	100% (100-100)	75.8% (65.4-86.1)	84.0% (83.7-84.3)

OMT, Other Microbiological Testing.

CRS, Composite Reference Standard.

mNGS, metagenomic Next-Generation Sequencing.

mNPS, metagenomic Nanopore Sequencing.

TB, tuberculosis group.

Non-TB, non-tuberculosis group.

PPV, Positive Predictive Value.

NPV, Negative Predictive Value.

**Table 3:** FPR and FNR of culture, OMT, mNGS and mNPS in TB and non-TB patients.

Test	TB		Non-TB		FPR (95%CI)	FNR (95%CI)
	+	-	+	-		
Culture	23/54	31/54	0/62	62/62	0% (0-0)	57.4% (45.6-72.2)
OMT	21/54	33/54	0/62	62/62	0% (0-0)	61.1% (49.4-75.6)
mNGS	17/29	12/29	1/31	30/31	3.2% (1.6-6.6)	41.4% (21.6-66.3)
mNPS	34/50	16/50	0/51	51/51	0% (0-0)	32.0% (21.4-47.9)

FPR, False positive rate; FNR, False negative rate.

### 3.3 Analysis of pathogens detected by mNGS, mNPS and culture

Of all the 120 cases, 45 pathogens were detected by the mNGS, 38 pathogens by mNPS and 5 pathogens by culture. In the mNGS platform, the top five pathogens were *Human herpes virus type 5, Torque teno virus, Human herpes virus type 4, Pneumocystis jirovecii*, and *Mycobacterium tuberculosis.* In mNPS platform, *Human herpesvirus type 5, Mycobacterium tuberculosis, Epstein-Barr virus, and Pneumocystis jirovecii (Human herpesvirus type 4*, Tied for fifth*)* were the top five pathogens, respectively (**Figure 2A**). In terms of the species of pathogens, The proportion of bacteria was higher than fungi and virus & mycoplasma in mNGS platform, while the bacteria and fungi were equally prevalent in NPS platform (**Figures 2B, C**). As a result of the methodology limitation, we only found five pathogens (*Mycobacterium tuberculosis, Bacillus marneffei, Staphylococcus aureus, Klebsiella pneumoniae and Streptococcus pneumoniae*) on culture platform (**Supplementary Figures S1**, **S2**). These results suggested that HIV patients primary co-infection of pathogens could be detected accurately through the mNGS and mNPS platforms., compared with culture platform. However, some pathogens weren’t detected by mNGS or mNPS, which might be due to the differences in the database.

### 3.4 Analysis of concurrent pathogens of HIV-infected patients in the TB group and non-TB group

**Figure 3:**
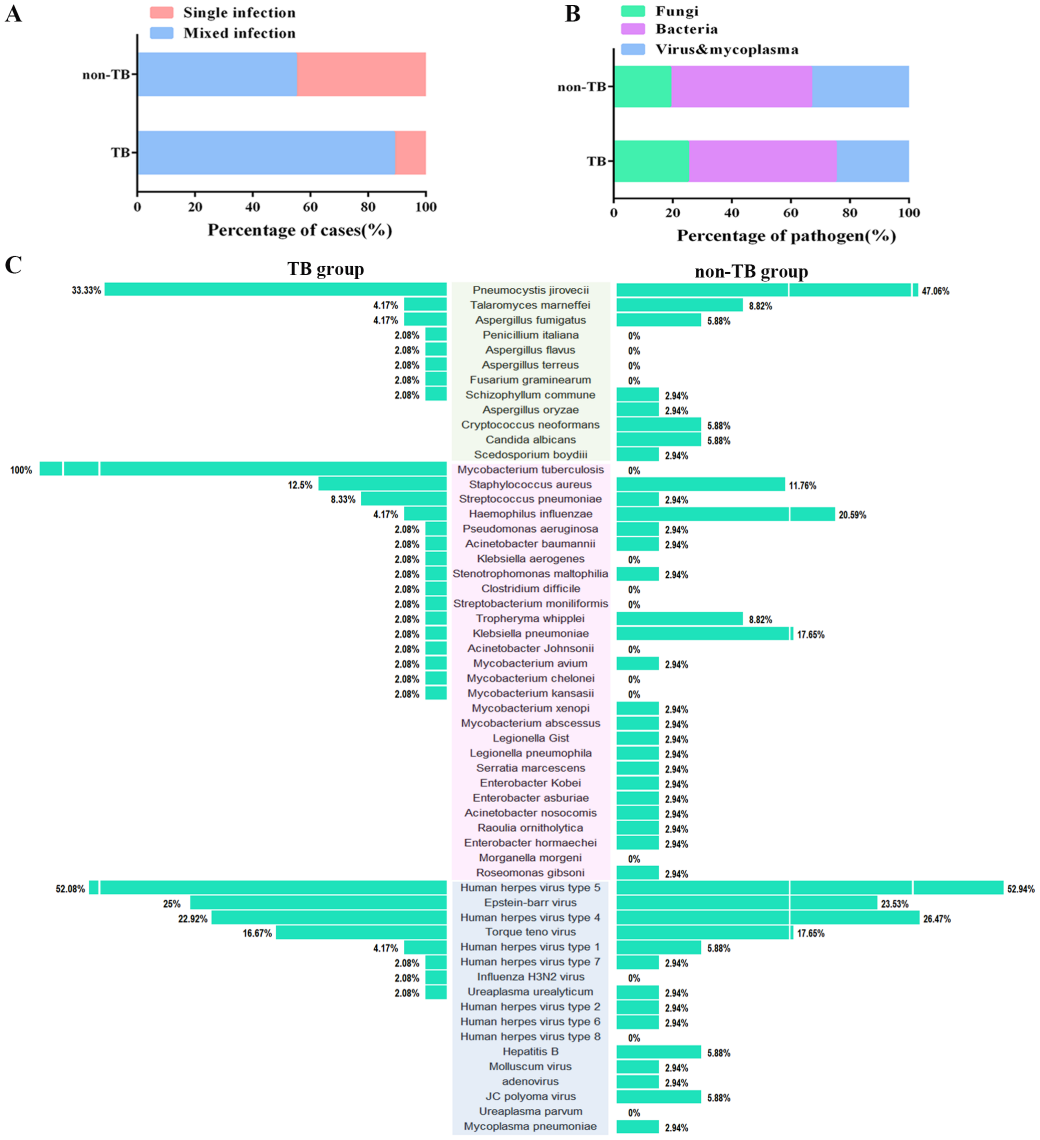
Distribution of the three categories of the clinical pathogen (fungi, bacteria, and viruses & mycoplasma) in TB and non-TB groups of HIV patients. **(A)** Stacked bar plots show the proportion of HIV patients infected with mixed and single pathogens in TB and non-TB groups. **(B)** Stacked bar plots illustrate the proportion of the three categories of clinical pathogen in TB and non-TB groups. **(C)** The butterfly diagram shows all of the pathogen-detected rates in TB and non-TB groups. Pathogen categories: Fungi, green background; Bacteria, pink background; Viruses & mycoplasma, blue background.

Of all the 120 cases, 82 cases suffered mixed infections. The proportion of mixed-infection cases (48/54, 88.9%) in the TB group was higher than those in the non-TB group (34/62, 54.8%) (**Figure 3A**), which may account for the patients with HIV-TB co-infection have lower immunity and were more likely to be infected by multiple pathogens than those infected with HIV alone. The HIV-TB co-infection patients were more susceptible to fungi infection, while the patients in non-TB group were more susceptible to viruses and mycoplasma (**Figure 3B**).

For concurrent pathogens detection, *Pneumocystis yersoni* (33.33% vs 47.06%), *Talaromyces marneffei* (4.17% vs 8.82%)*, Aspergillus fumigatus* (4.17% vs 5.88%) were the top three fungi both in TB and non-TB group*. Human herpesvirus type 5* (52.08% vs 52.94%), *Epstein-Barr virus* (25% vs 23.53%), and *Human herpesvirus type 4* (22.92% vs 26.47%) were the top three viruses detected both in the TB group and non-TB group. In terms of bacteria, *Staphylococcus aureus* (12.5%)*, Streptococcus pneumoniae* (8.33%), and *Haemophilus influenza* (4.17%) were the top three bacteria in TB group, whereas the top three bacteria in non-TB were *Haemophilus influenza* (20.59%), *Klebsiella pneumoniae* (17.66%), and *Streptococcus pneumoniae* (11.76%) (**Figure 3C**). The results showed that the main types of co-infected fungi and viruses in TB patients were consisted with the non-TB patients, while the types of bacteria in TB group were different with non-TB group. *Staphylococcus aureus* and *Streptococcus pneumoniae* were usually concurrent in HIV-TB co-infected patients.

### 3.5 Adjustments of antimicrobial therapy and outcome after mNGS and mNPS

**Figure 4:**
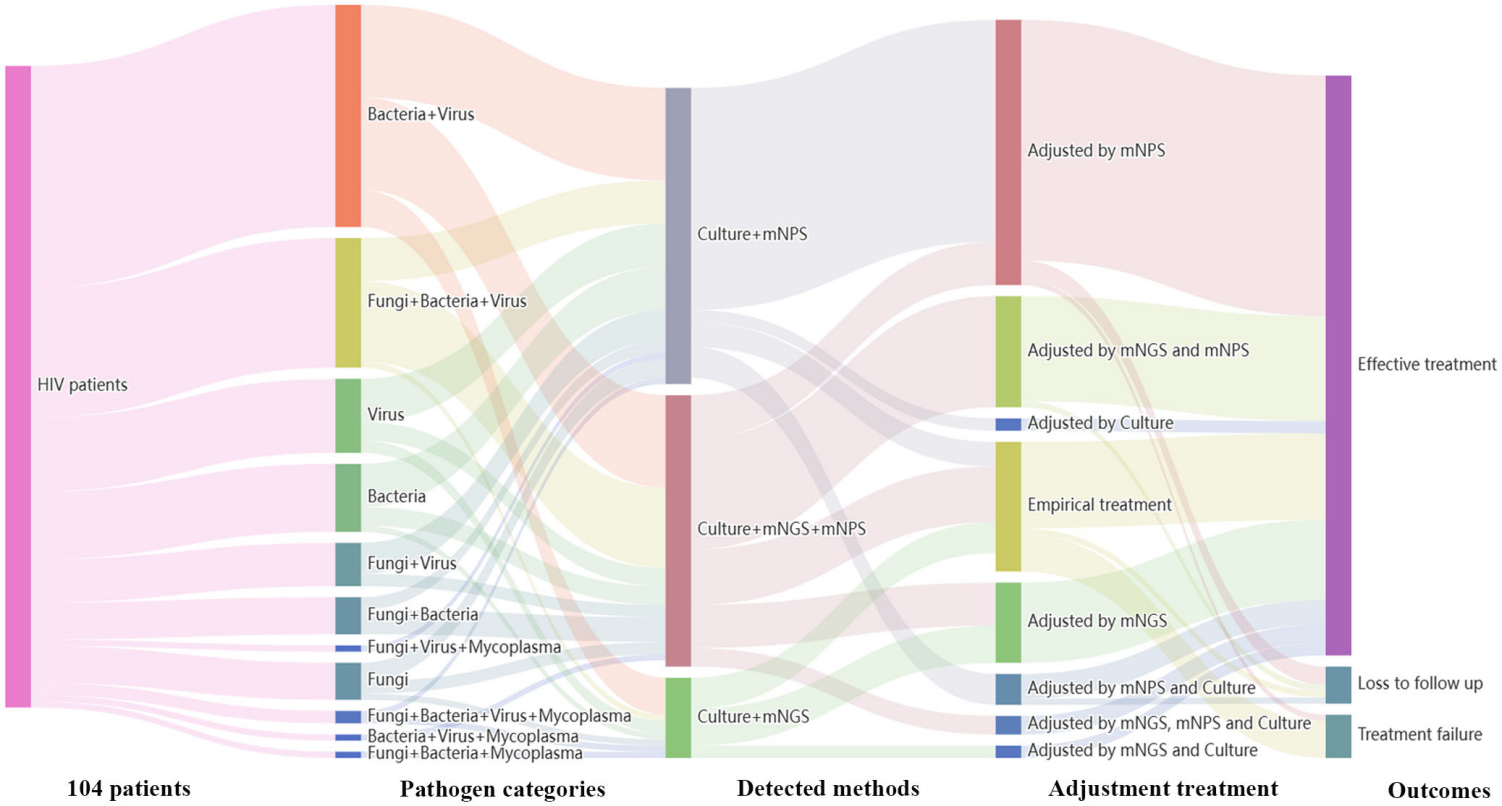
Sankey diagram demonstrated the treatment adjustment and outcomes based on mNPS and mNGS in HIV-infected patients. There are 104 patients were infected with other pathogens in addition to HIV. These patients were further classified by pathogen categories, detected methods, adjustment treatment, and outcomes. There are 69 patients (69/91, 75.8%) received the mNPS-directed medication adjustment and 63 patients (63/91, 69.2%) achieved effective treatment. There are 36 patients (36/57, 63.2%) of all the 57 patients modified the treatment strategy based on the positive mNGS results, and 35 patients (35/57, 61.4%) were treated effectively.

Treatment adjustments were made based on the clinical comprehensive diagnosis. Among the 120 HIV-infected patients, 104 patients were infected with one or more pathogens in addition to HIV. The results showed that 36 patients (36/104, 34.6%) were co-infected with Bacteria and Virus, 21 patients (21/104, 20.2%) were co-infected with Fungi, Bacteria and Virus. Among all the 91 cases detected by mNPS, 69 patients (69/91, 75.8%) received the mNPS-directed medication adjustment and 63 patients (63/91, 69.2%) achieved effective treatment. Meanwhile, 36 patients (36/57, 63.2%) of all the 57 patients modified the treatment strategy based on the positive mNGS results, and 35 patients (35/57, 61.4%) were treated effectively. Notably, of all the 18 patients that received empirical treatment, 14 patients (14/18, 77.8%) received effective treatment. However, 3 patients (3/18, 16.7%) were treated for failure based on the empirical treatment, while only one patient (1/91, 1.1%) failed to receive therapeutic benefits based on the mNPS platform (**Figure 4**).

## 4 Discussion

In HIV-infected patients, *Mycobacterium tuberculosis* is a common opportunistic pathogen that is widely distributed worldwide. HIV-infected patients complicated with tuberculosis (TB) will accelerate the course of disease progress, which greatly increases the death rate of patients. Therefore, accurate and timely TB diagnosis is essential for treatment decisions of HIV-infected patients and disease management. The mNGS and mNPS are powerful and promising technique with high specificity and sensitivity for rapid pathogens detection in recent years. To date, no studies have directly evaluated the performances of mNGS and mNPS for TB diagnosis of HIV-patients. Mao et al. found the mNGS showed excellent performance in mixed infection in HIV-patients, compared with culture (Zhang et al., 2024). Lin et al. investigated the detected performance of traditional methods and targeted NPS for the diagnosis of pneumonia (Lin et al., 2024), and it is also reported that mNPS of infectious fluid is a promising supplement for gold-standard culture in real-world clinical scenario (Zhao et al., 2024). In this paper, we firstly evaluated the diagnostic performance of mNGS, mNPS and traditional methods for rapid tuberculosis (TB) diagnosis in HIV-positive patients, which is not reported before. We found mNGS and mNPS have higher sensitivity and specificity compared to traditional microbiological methods, and these advanced sequencing techniques enable timely and accurate adjustments to treatment, leading to improved patient outcomes.

Traditional microbiological tests such as culture, smear microscopy, serology, and antigen detection, are routinely performed strategies in clinical diagnosis. However, these methods have low time efficiency and only a limited number of pathogens can be detected, which restricts the development of personalized precision medication (Ewig et al., 2002; Schlaberg et al., 2017). David et al. showed the mNGS and Targeted NGS workflows have high performance for detection of respiratory mixed pathogens (Gaston et al., 2022). In our study, 68.2% of HIV-infected patients were diagnosed with mixed infection by the mNGS or mNPS platform, especially for the TB group, whereas none of the complete spectrum of pathogens of the mixed-infected patients was detected by culture or other traditional methods. Therefore, the mNGS and mNPS showed apparent and effective advantages in detecting mixed infection, especially for HIV-infected patients who have lower immunity.

HIV-infected patients are commonly accompanied by respiratory symptoms such as cough, purulent sputum, breathlessness, or dyspnea, which are caused by LRTIs. It is reported that HIV patients are more likely to develop LRTIs (De Munter et al., 2017). The most common pathogens of LRTIs for HIV-infected patients like *Mycobacterium tuberculosis, Pneumocystis Jiroveci*, *Herpes virus*, and *Cytomegalo virus*. In our study, we found that *Mycobacterium tuberculosis, Human herpes virus type 5 (Cytomegalo virus), Torque teno virus, Human herpes virus type 4, Epstein-Barr virus, Pneumocystis jirovecii* are the main pathogens in HIV patients, which is consistent with the previous study.

Our study also has some limitations. Firstly, the sample size is limited and the number of patient samples tested by the three detection methods varied significantly, which could affect the stability and reliability of the statistic. A larger and prospective cohort is needed to verify our conclusion. Secondly, our study was a single-center study and may not represent the infectious spectrum of all HIV-infected patients. Third, since the pathogen spectrum of mNPS and mNGS is broad, some non-pathogenic microorganisms such as colonizing species could also detected. Failure to properly interpret the testing results may lead to erroneous disease treatment. Therefore, it is essential to make an accurate diagnosis based on clinical testing, clinical symptoms, inflammatory factors, and imaging evidence.

In conclusion, our study indicated that mNPS and mNGS have high sensitivity and specificity for TB diagnosis compared with the traditional methods, and mNPS seems to have the best diagnostic performance. Moreover, mNGS and mNPS showed apparent advantages in detecting mixed infection, the mNPS and mNGS-directed medication adjustment have effective treatment outcomes for HIV-infected patients who have lower immunity.

## Data Availability

The datasets presented in this study have been deposited into CNGB Sequence Archive (CNSA) of China National GeneBank DataBase (CNGBdb) with accession number CNP0005739.

## Supplementary material

Supplementary Figure 1 Comparison of the pathogens detection rate of HIV-infected patients on mNGS platform and Culture. Pathogen categories: Fungi, dark blue background; Bacteria, pink background; Virus & mycoplasma, light blue background.

Supplementary Figure 2 Comparison of the pathogens detection rate of HIV-infected patients on mNPS platform and Culture. Pathogen categories: Fungi, dark blue background; Bacteria, pink background; Virus & mycoplasma, light blue background.
