# Supplementary figures and images for: Performance of metagenomic Next-Generation Sequencing and metagenomic Nanopore Sequencing for the diagnosis of tuberculosis in HIV-positive patients
Source: Front Cell Infect Microbiol. 2024 Aug 21;14:1423541. doi: 10.3389/fcimb.2024.1423541 (PMC11371759; doi:10.3389/fcimb.2024.1423541)

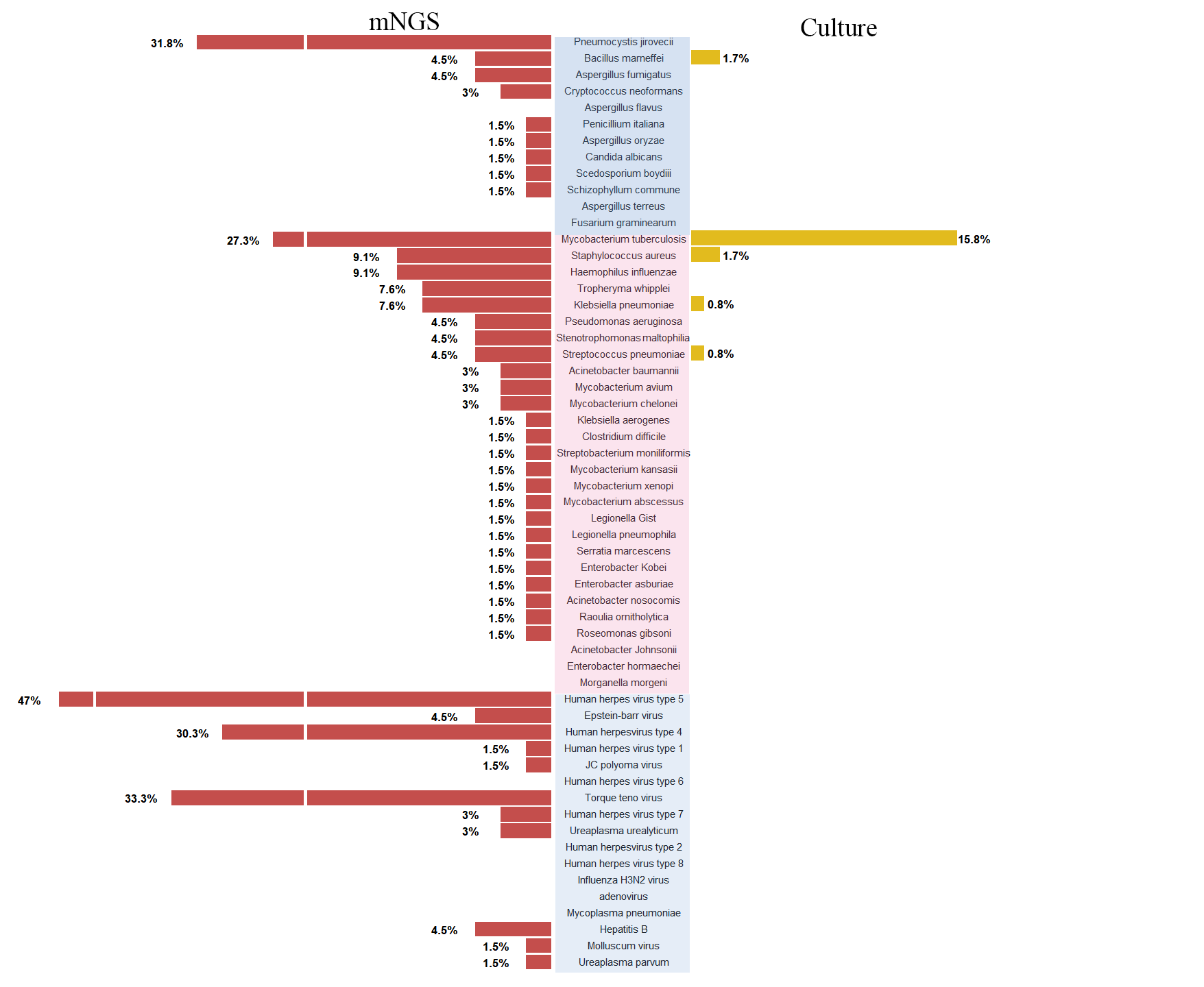

Supplement: Supplementary Figure 1 — Comparison of the pathogens detection rate of HIV-infected patients on mNGS platform and Culture. Pathogen categories: Fungi, dark blue background; Bacteria, pink background; Virus & mycoplasma, light blue background. [file Image1.tif]

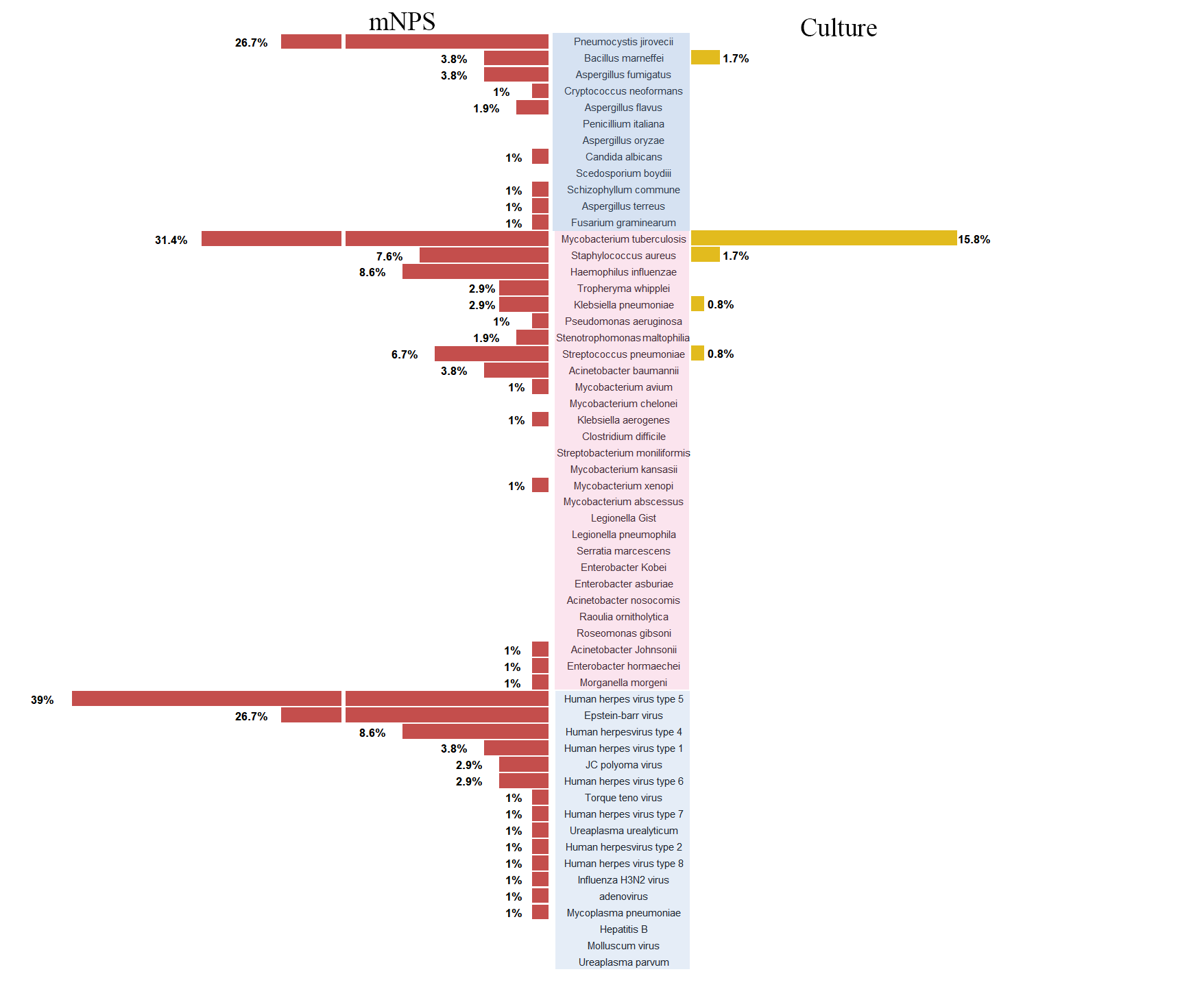

Supplement: Supplementary Figure 2 — Comparison of the pathogens detection rate of HIV-infected patients on mNPS platform and Culture. Pathogen categories: Fungi, dark blue background; Bacteria, pink background; Virus & mycoplasma, light blue background. [file Image2.tif]
